# Risk Models and Scoring Systems for Predicting the Prognosis in Critically Ill Cirrhotic Patients with Acute Kidney Injury: A Prospective Validation Study

**DOI:** 10.1371/journal.pone.0051094

**Published:** 2012-12-07

**Authors:** Heng-Chih Pan, Chang-Chyi Jenq, Ming-Hung Tsai, Pei-Chun Fan, Chih-Hsiang Chang, Ming-Yang Chang, Ya-Chung Tian, Cheng-Chieh Hung, Ji-Tseng Fang, Chih-Wei Yang, Yung-Chang Chen

**Affiliations:** 1 Kidney Research Center, Department of Nephrology, Chang Gung Memorial Hospital, Taipei, Taiwan; 2 Division of Gastroenterology, Chang Gung Memorial Hospital, Taipei, Taiwan; 3 Chang Gung University College of Medicine, Taoyuan, Taiwan; Beth Israel Deaconess Medical Center, United States of America

## Abstract

**Background:**

Cirrhotic patients with acute kidney injury (AKI) admitted to intensive care units (ICUs) show extremely high mortality rates. We have proposed the MBRS scoring system, which can be used for assessing patients on the day of admission to the ICU; this new system involves determination of mean arterial pressure (MAP) and bilirubin level and assessment of respiratory failure and sepsis. We had used this scoring system to analyze the prognosis of ICU cirrhotic patients with AKI in 2008, and the current study was an external validation of this scoring system.

**Methods:**

A total of 190 cirrhotic patients with AKI were admitted to the ICU between March 2008 and February 2011. We prospectively analyzed and recorded the data for 31 demographic parameters and some clinical characteristic variables on day 1 of admission to the ICU; these variables were considered as predictors of mortality.

**Results:**

The overall in-hospital mortality rate was 73.2% (139/190), and the 6-month mortality rate was 83.2% (158/190). Hepatitis B viral infection (43%) was observed to be the cause of liver disease in most of the patients. Multiple logistic regression analysis indicated that the MBRS and Acute Physiology and Chronic Health Evaluation III (ACPACHE III) scores determined on the first day of admission to the ICU were independent predictors of in-hospital mortality in patients. In the analysis of the area under the receiver operating characteristic (AUROC) curves, the MBRS scores showed good discrimination (AUROC: 0.863±0.032, *p*<0.001) in predicting in-hospital mortality.

**Conclusion:**

On the basis of the results of this external validation, we conclude that the MBRS scoring system is a reproducible, simple, easy-to-apply evaluation tool that can increase the prediction accuracy of short-term prognosis in critically ill cirrhotic patients with AKI.

## Introduction

Liver cirrhosis is characterized by disturbances in the systemic circulation, including marked arterial vasodilation that occurs principally in the splanchnic circulation, reduces the total peripheral vascular resistance and arterial pressure, and causes a secondary increase in the cardiac output. These abnormalities are central to the development of several major complications in patients with cirrhosis, such as the hepatorenal syndrome, ascites, spontaneous bacterial peritonitis, dilutional hyponatremia, and hepatopulmonary syndrome. Renal failure is the most clinically relevant condition among these conditions because its appearance generally indicates a very poor prognosis [1]–[10].

We developed the MBRS scoring system, a simple prognostic model that includes determination of mean arterial pressure (MAP) and serum bilirubin level and assessment of acute respiratory failure and sepsis. These 4 variables are to be analyzed on day 1 of admission to the intensive care unit (ICU). We used this model to analyze and predict the in-hospital mortality in 111 critically ill cirrhotic patients with acute kidney injury (AKI) [11]. The MBRS score [calculated using the following predictors: MAP, <80 mmHg; serum bilirubin level, >80 µmol/L (4.7 mg/dl); acute respiratory failure, and sepsis] was defined as the sum of the values of the individual predictors, each value ranging from 0 to 4. This score has better discriminatory power than the other evaluation systems such as the Child-Pugh [12], model for end-stage liver disease (MELD) [13], Acute Physiology and Chronic Health Evaluation II and III (APACHE II & III) [14], [15], and sequential organ failure assessment (SOFA) system [16]. The area under the receiver operating characteristic curve (AUROC) values for the MBRS scores were significantly more than the AUROC values plotted for the Child-Pugh and APACHE II scores [11].

The prognostic value of MBRS scores for cirrhotic patients with AKI admitted to ICUs needs to be validated further through studies on different cohorts. Further confirmation is particularly important because we observed that, over time, the mortality rates of patients who showed the same characteristics at admission typically decreased. Possible causes that may not have affected the scoring variables, including improvements in therapies and management of bleeding, renal failure, respiratory failure, and sepsis, require additional testing in new study cohorts [2], [17]. To the best of our knowledge, no prospective clinical study has validated predictive power of MBRS scores on critically ill cirrhotics with AKI. We aimed to evaluate the reproducibility of the MBRS scoring system in predicting the in-hospital mortality rate by performing an external validation.

## Materials and Methods

### Ethics statement

This clinical study was conducted in full compliance with the ethical principles of the Declaration of Helsinki and was consistent with Good Clinical Practice guidelines and applicable local regulatory requirements. The local institutional review board of Chang Gung Memorial Hospital approved our study protocol. Patients meeting the inclusion criteria were invited to participate in this study on their first day of ICU admission. Trained physicians evaluated their mental status during the screening and informed consent procedure. Written informed consent was obtained from all mentally competent patients or next-of-kin of compromised ones prior to their participation.

### Patient information and data collection

This study was performed between March 2008 and February 2011 in a 10-bed specialized ICU (hepatogastroenterology ICU) at a 2000-bed tertiary care referral hospital in Taiwan. In this study, we included 190 consecutive patients with hepatic cirrhosis and AKI requiring intensive monitoring and/or treatment that cannot be provided outside the ICU. We excluded patients who did not match the criteria of AKI (127 patients), patients who had previous end-stage renal disease patients undergoing regular renal replacement therapy (38 patients); patients whose hospital stay length <24 h (30 patients), patients who had received liver transplantation (16 patients), and patient who were readmitted (21 patients).

The following data were collected prospectively: demographic data; reason for admission to the ICU; immediate diagnosis; severity of the illness; MELD, SOFA, APACHE II, and APACHE III scores determined on the first day of ICU admission; the duration of hospitalization; and the treatment outcome. The primary study outcome was the in-hospital mortality rate. Follow-up examinations were performed 6 months after discharge of the patients from the hospital by analyzing the chart records.

### Definitions

Cirrhosis was diagnosed on the basis of the results of liver histology or a combination of physical signs and symptoms and findings from biochemical analysis and ultrasonography. Acute kidney injury was defined as a 50% increase in serum creatinine (SCr) level or an immediate requirement for renal replacement therapy. The measurement of SCr levels was repeated following the withdrawal of diuretics in the patients. A study stated that a 50% increase in SCr levels indicates acute renal dysfunction as per the RIFLE (risk of renal failure, injury to the kidney, failure of kidney function, loss of kidney function, and end-stage renal failure) classification system. In that study, the patient had RIFLE-R stage disease since the patient's SCr level had increased by a factor of 1.5 or more from the baseline [18]. Baseline SCr was the first value measured during hospitalization. The modification of diet in renal disease (MDRD) formula was used to estimate the baseline SCr levels in 15 patients because these patients had been admitted directly to the ICU and their previous SCr levels were unknown [18]. Respiratory failure was defined as a respiratory rate of ≤5/min or of ≥50/min, and/or requirement of mechanical ventilation for ≥3 days, and/or fraction of inspired oxygen (FiO_2_) of >0.4, and/or a positive end-expiratory pressure of >5 cm H_2_O [19]–[21]. Sepsis was defined as systemic inflammatory response syndrome (SIRS) plus suspected or proven infection. According to the guidelines of the American College of Chest Physician/Society of Critical Care Medicine (ACCP/SCCM) Consensus Conference, SIRS was defined as patients with more than one of the following clinical findings: body temperature, >38°C or <36°C; heart rate, >90 beats per minute; hyperventilation evidenced by a respiratory rate of >20 cycles per minute or a Paco2 of <32 mm Hg; and a white blood cell count of >12,000 cells per µL or <4,000 cells per µL [22].

The severity of the liver disease on admission to the ICU was determined by using the Child–Pugh and MELD scoring systems. Severity of the illness can also be assessed by using the SOFA, APACHE II, and APACHE III scoring systems. The MBRS score was based on 4 independent prognostic predictors: lower threshold of MAP, i.e., 80 mmHg (1 point); upper threshold cut-off of serum bilirubin, i.e., 80 µmol/L or 4.7 mg/dl (1 point); acute respiratory failure (1 point); and sepsis (1 point). Assessment of these predictors was performed on the day 1 of admission to the ICU [11]. The worst physiological and biochemical values determined on the first day of ICU admission were recorded. Clinical management of these patients was done by the method described elsewhere [11].

### Clinical management

All patients received careful history taking, physical examination and a number of laboratory measurements. Potential nephrotoxins were discontinued. Renal ultrasound was arranged to exclude postrenal azotemia on the first day of ICU admission.

Patients who had a clear history of septic or hypovolemic shock, or a recent history of nephrotoxins exposure with high UNa (>40 mEq/L), high FENa (2%), and urine osmolality under 350 mOsm/kg were treated as intrinsic azotemia as further described. Patients with upper gastrointestinal bleeding from esophageal varices were initially treated with emergency sclerotherapy and administration of vasopressors. Patients with peptic ulcer, either with active bleeding, visible vessels or visible clots, were treated with sclerosing agents, followed by proton pump inhibitors. All patients received intravenous fluid depending on their fluid volume and electrolyte status. The decision to transfuse packed red blood cells (PRBC) was made according to the criteria of the attending physician or whenever a patient's hemoglobin level dropped below 8 g/dL [23]. Patients with bacterial infections on admission and patients who developed bacterial infections during hospitalization were treated with appropriate empiric antibiotic therapy according to culture results and the results of appropriate diagnostic methods. When acute renal failure was severe or progressive and measures to improve renal function had been unsuccessful, renal replacement therapy was performed [4].

In all other patients, diuretics, lactulose, and vasodilators were not given. Volume expansion therapy such as intravenous albumin (1 g/Kg QD or BID, up to a maximum of 100 g) and/or artificial plasma expanders were administrated to correct volume depletion and to keep central venous pressure over 10 cmH2O every 12 hrs for 2 days. Daily measurements of urine output and serum creatinine began on day 1 of ICU admission and continued for at least 2 days. Patients with volume-responsive serum creatinine improvement was treated as prerenal azotemia and kept receiving volume supply [4].

Patients without volume-responsive acute kidney injury who had no shock, recent nephrotoxin exposure nor evidence of parenchymal kidney disease history (by urinalysis and image) were treated as hepatorenal syndrome with terlipressin (0.5–2 mg iv every 4–6 hrs) plus albumin for at least 3 days. Others were treated as intrinsic azotemia as described above [4].

### Statistical analysis

Descriptive statistics were expressed as mean and standard deviation values unless otherwise stated. In the primary analysis, we compared the number of hospital survivors with the number of nonsurvivors. Normal distribution of all the variables was analyzed using the Kolmogorov–Smirnov test. Student's *t*-test was used to compare the mean values of continuous variables and normally distributed data; in the case of the other data, the Mann–Whitney *U* test was used. Categorical data were analyzed using the χ^2^ test. The chi-square test for trends were used to assess categorical data associated with MBRS scores. Correlation of paired-group variables were assessed using linear regression and Pearson analysis. We assessed the risk factors for in-hospital mortality by using univariate analysis, and the variables that were found to be statistically significant (*p*<0.05) in the univariate analysis were included in the multivariate analysis. A multiple logistic regression model and forward elimination of data were used to analyze these variables.

Calibration was assessed using the Hosmer–Lemeshow goodness-of-fit test to compare the number of observed deaths with the number of predicted deaths in the risk groups for the entire range of death probabilities. Discrimination was calculated using the AUROC values. The AUROC values were compared using a nonparametric approach. The AUROC analysis was also utilized to calculate the cut-off values, sensitivity, specificity, and overall correctness. Finally, cut-off points were calculated by calculating the best Youden index (sensitivity+specificity−1). Cumulative survival curves as a function of time were plotted using the Kaplan–Meier approach and were compared using the log rank test. All the statistical tests were 2-tailed. A *p* value of *<*0.05 was considered statistically significant. The data were analyzed using the Statistical Analysis for Social Sciences software, version 12.0 for Windows (SPSS, Inc., Chicago, IL, USA).

## Results

### Subject characteristics

A total of 190 cirrhotic patients with AKI treated at the specialized hepatogastroenterology ICU were enrolled in the study between March 2008 and February 2011. The overall in-hospital mortality rate for the entire group was 73.2% (139/190), and the 6-month mortality rate was 83.2% (158/190). The demographic data and clinical characteristics of both the survivors and the nonsurvivors are listed in table 1. The median age of the patients was 58 years; 141 patients were men (74%), and 49 were women (26%). The median duration of stay in the ICU was 9 days. The causes of cirrhosis, the reasons for admission to the ICU, and presumptive etiologies of AKI are listed in table 2. Hepatitis B viral infection was observed to the cause of liver diseases in most of the patients. The most frequent reason for admission to the ICU was upper gastrointestinal bleeding. Patients who developed AKI tended to have a history of infection.

**Table 1:** Patients' demographic data and clinical characteristics.

	All patients (n = 190)	Survivors (n = 51)	Non-survivors (n = 139)	*p*-value
Age (years)	58±1	58±2	59±1	NS (0.738)
Gender (M/F)	141/49	41/10	100/39	NS (0.238)
Length of ICU stay (days)	9±9	6±4	10±11	<0.001
Length of hospital stay (days)	25±25	32±33	23±21	0.067
Serum Creatinine, ICU first day (mg/dL)	3.2±2.4	2.5±2.2	3.6±2.4	0.005
MAP, ICU admission (mmHg)	73±18	86±16	69±17	<0.001
Glasgow coma scale, ICU admission	9±5	10±5	9±5	NS (0.104)
Leukocytes, ICU first day (g/dL)	12.8±8.0	10.0±5.8	13.9±8.5	0.001
Haemoglobin, ICU first day (g/dL)	9.2±2.2	9.0±1.9	9.2±2.4	NS (0.480)
Albumin, ICU first day (g/dL)	2.5±0.5	2.6±0.6	2.4±0.5	NS (0.130)
Sodium, ICU first day (mmol/L)	135±17	138±9	134±19	NS (0.151)
Bilirubin, ICU first day (umol/L) [median]	11.2 [5.4]	4.6 [3.1]	13.6 [8.7]	<0.001
Prothrombin time INR, ICU first day [median]	2.8 [2.3]	1.9 [1.6]	3.1 [2.3]	0.002
AST, ICU first day (units/L) [median]	530 [94]	133 [67]	678 [100]	0.008
ALT, ICU first day (units/L) [median]	182 [45]	56 [32]	228 [53]	0.002
Platelets, ICU first day (×10^9^/L) [median]	95 [73]	91 [73]	97 [69]	NS (0.645)
DM (Yes/No)	52/138	14/37	38/101	NS (0.988)
Previous ascites (Yes/No)	94/96	20/31	74/65	NS (0.087)
Previous SBP (Yes/No)	39/150	8/43	31/107	NS (0.307)
Previous hepatic encephalopathy (Yes/No)	116/74	29/22	87/52	NS (0.473)
Previous EV bleeding (Yes/No)	86/104	23/28	63/76	NS (0.978)
Previous peptic ulcer bleeding (Yes/No)	59/131	17/34	42/97	NS (0.681)
Previous hepatoma (Yes/No)	59/131	10/41	49/90	0.039
Previous renal failure (Yes/No)	57/133	17/34	40/99	NS (0.544)
Respiratory failure, ICU first day (Yes/No)	37/153	4/47	33/106	0.014
Sepsis, ICU admission (\Yes/No)	71/119	11/40	60/79	0.006
Child-Pugh points (mean± SD)	11.8±2.1	11.0±2.4	12.0±2.0	0.036
MELD score (mean ± SD)	33.2±1.1	24.7±8.8	35.8±11.3	<0.001
APACHE II (mean ± SD)	25.5±0.77	20.9±6.9	26.9±8.5	0.001
APACHE III (mean ± SD)	106.0±3.19	77.9±29.1	114.7±32.6	<0.001
SOFA (mean ± SD)	11.6±0.3	8.06±2.8	12.9±3.7	<0.001

Abbreviation: M, male; F, female; ICU, intensive care unit; MAP, mean arterial pressure; INR, international normalized ratio; AST, aspartate aminotransferase; ALT, alanine aminotransferase; DM, diabetes mellitus; SBP, spontaneous bacterial peritonitis; EV, esophageal varices; SD, standard derivation; NS, not significant; MELD, model for end-stage liver disease; APACHE, acute physiology and chronic health evaluation; SOFA, sequential organ failure assessment.

**Table 2:** Causes of cirrhosis, reasons for ICU admission and presumptive causes of AKI.

	All patients (%)	Survivors (%)	Non-survivors (%)	*p*
***Causes of cirrhosis***				
Alcoholic	33 (17)	15 (29)	18 (13)	0.005
Hepatitis B	60 (32)	6 (12)	54 (39)	<0.001
Hepatitis C	39 (20)	11 (22)	28 (20)	NS (0.716)
Alcoholic+Hepatitis B	14 (7)	8 (16)	6 (4)	0.006
Alcoholic+Hepatitis C	3 (2)	1 (2)	2 (1)	NS (0.771)
Hepatitis B+Hepatitis C	5 (3)	1 (2)	4 (3)	NS (0.755)
Alcoholic+Hepatitis B+Hepatitis C	1 (1)	0 (0)	1 (1)	NS (1.000)
Other causes^a^	35 (17)	9 (18)	26 (19)	NS (0.868)
***Primary ICU admission***				
Severe UGI bleeding	46 (24)	18 (35)	28 (20)	NS (0.031)
Severe sepsis	34 (18)	5 (10)	29 (21)	NS (0.078)
Hepatic encephalopathy	25 (13)	11 (22)	14 (10)	0.038
Respiratory failure	10 (5)	3 (6)	7 (5)	NS (0.817)
AKI require renal replacement	11 (6)	2 (4)	9 (6)	NS (0.504)
Others^b^	64 (35)	12 (24)	52 (37)	NS (0.073)
***Presumptive etiology of AKI***				
Pre-renal failure	31 (16)	13 (25)	18 (13)	0.038
Infection-induced AKI	51 (27)	5 (10)	46 (33)	0.001
Parenchymal renal diseases	11 (6)	5 (10)	6 (4)	NS (0.151)
Acute tubular necrosis	17 (9)	3 (6)	14 (10)	NS (0.370)
Nephrotoxic acute renal failure	9 (5)	6 (12)	3 (2)	0.006
HRS type I/type II/total	10/17/27 (14)	1/2/3 (6)	9/15/24 (17)	0.046
Others^c^	44 (23)	16 (31)	28 (20)	NS (0.104)

Abbreviation: UGI, upper gastrointestinal; AKI, acute kidney injury; NS, not significant; ICU, intensive care unit; HRS, hepatorenal syndrome.

a Primary biliary cirrhosis, autoimmune hepatitis, and other unknown causes.

b Pancreatitis, hepatoma rupture, unknown cause, or multifactor related.

c Mixed type, unknown cause, or multifactor related.

### Risk factors for in-hospital mortality

The univariate analysis showed that 12 (Table 3) of the 31 variables (Table 1) were good prognostic indicators. On performing multivariate analysis, we identified that the MBRS and APACHE III scores determined on admission to the ICU have independent prognostic significance for assessing in-hospital mortality (Table 3). Regression coefficients of these variables were used to calculate the odds of death in each patient as follows:
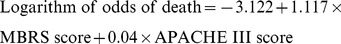



**Table 3:** Variables showing prognostic significance.

Parameter	Beta coefficient	Standard error	Odds ratios (95% CI)	*p*-value
***Univariate logistic regression***				
Length of ICU stay	0.086	0.033	1.090(1.022–1.164)	0.009
Length of hospital stay	−0.013	0.006	0.987(0.975–0.999)	0.038
Serum Creatinine, ICU first day	0.258	0.095	1.295(1.074–1.561)	0.007
MAP, ICU admission	−0.060	0.015	0.942(0.915–0.969)	<0.001
Leukocytes, ICU first day	<0.001	<0.001	1.000(1.000–1.000)	0.004
Bilirubin, ICU first day	0.123	0.032	1.131(1.063–1.204)	<0.001
Prothrombin time INR, ICU first day	0.555	0.235	1.742(1.099–2.762)	0.018
AST, ICU first day	0.002	0.001	1.002(1.000–1.003)	0.053
ALT, ICU first day	0.005	0.002	1.005(1.000–1.010)	0.041
Previous hepatoma	0.803	0.395	2.232(1.030–4.840)	0.042
Respiratory failure, ICU first day	1.297	0.558	3.658(1.226–10.913)	0.020
Sepsis, ICU admission	1.016	0.381	2.762(1.309–5.829)	0.008
Child-Pugh points	0.203	0.099	1.225(1.009–1.486)	0.040
MELD	0.119	0.029	1.127(1.065–1.192)	<0.001
APACHE II	0.099	0.031	1.104(1.038–1.174)	0.002
APACHE III	0.040	0.009	1.040(1.022–1.059)	<0.001
SOFA	0.453	0.078	1.573(1.351–1.831)	<0.001
***Multivariate logistic regression***				
MBRS score	1.117	0.359	3.059(1.527–6.448)	0.002
APACHE III	0.040	0.014	1.040(1.012–1.069)	0.004
Constant	−3.122	1.988	0.116	0.044

Abbreviation: ICU, intensive care unit; INR, international normalized ratio; MAP, mean arterial pressure; CI, confidence intervals; MELD, model for end-stage liver disease; APACHE, acute physiology and chronic health evaluation; SOFA, sequential organ failure assessment; MBRS, mean arterial pressure, bilirubin, respiratory failure and sepsis.

### Severity of illness scoring systems

We have listed the results of goodness-of-fit as measured by the Hosmer-Lemeshow χ^2^ statistic denoting the predicted mortality risk, the predictive accuracy of the Child-Pugh points, MBRS, MELD, APACHE II, III, and SOFA scores in table 4. The comparison between discriminatory values of the 7 scoring systems has also been included in table 4. The AUROC analysis showed that the MBRS score has the best discriminatory power. The discriminatory powers of the RIFLE classification, Child-Pugh and the APACHE II scores were significantly lower than that of the MBRS score.

**Table 4:** Calibration and discrimination for the scoring methods in predicting hospital mortality.

	Calibration			Discrimination		
	Goodness-of-fit (x^2^)	df	*p*	AUROC±SE	95% CI	*p*
***RIFLE-R (n = 68)***						
MBRS	3.349	3	0.341	0.810±0.077	0.660–0.961	0.001
SOFA	5.969	8	0.651	0.673±0.089	0.498–0.848	0.074
MELD	7.658	8	0.468	0.621±0.100	0.424–0.817	0.214
***RIFLE-I (n = 33)***						
MBRS	0.466	3	0.926	0.873±0.103	0.670–1.000	0.020
SOFA	2.234	8	0.973	0.845±0.099	0.650–1.000	0.031
MELD	3.504	6	0.743	0.764±0.123	0.522–1.000	0.100
***RIFLE-F (n = 89)***						
MBRS	1.193	2	0.551	0.933±0.031	0.872–0.994	<0.001
SOFA	2.939	8	0.938	0.911±0.042	0.828–0.994	<0.001
MELD	4.880	8	0.770	0.851±0.061	0.732–0.970	<0.001
***Overall (n = 190)***						
MBRS	1.160	3	0.763	0.863±0.032	0.801–0.925	<0.001
SOFA	5.342	8	0.721	0.848±0.029	0.791–0.906	<0.001
MELD	4.658	8	0.793	0.776±0.047	0.683–0.868	<0.001
Child-Pugh points	7.740	5	0.171	0.622±0.065*	0.496–0.749	0.047
APACHE II	4.574	8	0.802	0.686±0.053*	0.583–0.789	0.003
APACHE III	12.531	8	0.129	0.793±0.045	0.705–0.881	<0.001
RIFLE	0.329	1	0.566	0.679±0.043*	0.679–0.764	<0.001

Abbreviation: MBRS, mean arterial pressure, bilirubin, respiratory failure and sepsis; MELD, model for end-stage liver disease; APACHE, acute physiology and chronic health evaluation; SOFA, sequential organ failure assessment; df, degree of freedom; RIFLE, risk of renal failure, injury to kidney, failure of kidney function, loss of kidney function, and end-stage renal failure; AUROC, areas under the receiver operating characteristic curve; SE, standard error; CI, confidence intervals; NS, not significant.

* *p*<0.05 versus MBRS score.

We examined the correlation between the scores determined by the Child-Pugh points, MBRS, MELD, APACHE II, III, and SOFA systems. The correlations between the scoring systems used on the first day of admission of the patients to the ICU have been listed in table 5. The MBRS score showed positive correlations with other scores in terms of the likelihood of in-hospital mortality (r>0.25, *p*<0.01) (Table 5).

**Table 5:** Correlation between scoring systems on the first day of ICU admission (Spearman rank correlation coefficients: r).

Scores	MBRS	MELD	APACHE II	APACHE III	SOFA
Child-Pugh points	0.308**	0.436**	0.048	0.231*	0.357**
MBRS	-	0.450**	0.239**	0.375**	0.573**
MELD		-	0.141	0.372**	0.536**
APACHE II			-	0.682**	0.530**
APACHE III				-	0.693**

Abbreviation: MBRS, mean arterial pressure, bilirubin, respiratory failure and sepsis; MELD, model for end-stage liver disease; APACHE, acute physiology and chronic health evaluation; SOFA, sequential organ failure assessment.

* *p*<0.05;

** *p*<0.01.

To assess the validity of the applied scoring methods, the sensitivity, specificity, and overall correctness of the prediction at selected cut-off points that provided the best Youden index were analyzed, and this data is listed in table 6. The MBRS score had the best Youden index and the highest overall correctness of prediction.

**Table 6:** Prediction of subsequent hospital mortality on the first day of ICU admission.

Predictive factors	Cutoff point	Youden index	Sensitivity (%)	Specificity (%)	Overall correctness (%)
MAP (mmHg)	80^a^	0.41	62	79	71
Bilirubin(umol/L)	80^a^	0.47	68	78	73
Respiratory failure	Yes^a^	0.16	24	92	58
Sepsis	Yes^a^	0.22	43	78	61
MBRS score	2^a^	0.57	68	88	78
Child-Pugh points	11^a^	0.29	67	62	65
MELD score	34^a^	0.39	49	90	79
APACHE II	25^a^	0.31	52	79	66
APACHE III	88^a^	0.51	82	69	76
SOFA	9^a^	0.53	82	71	76

Abbreviation: MAP, mean arterial pressure; ICU, intensive care unit; MBRS, mean arterial pressure, bilirubin, respiratory failure and sepsis; MELD, model for end-stage liver disease; APACHE, acute physiology and chronic health evaluation; SOFA, sequential organ failure assessment.

a Value giving the best Youden index.

**Figure 1:**
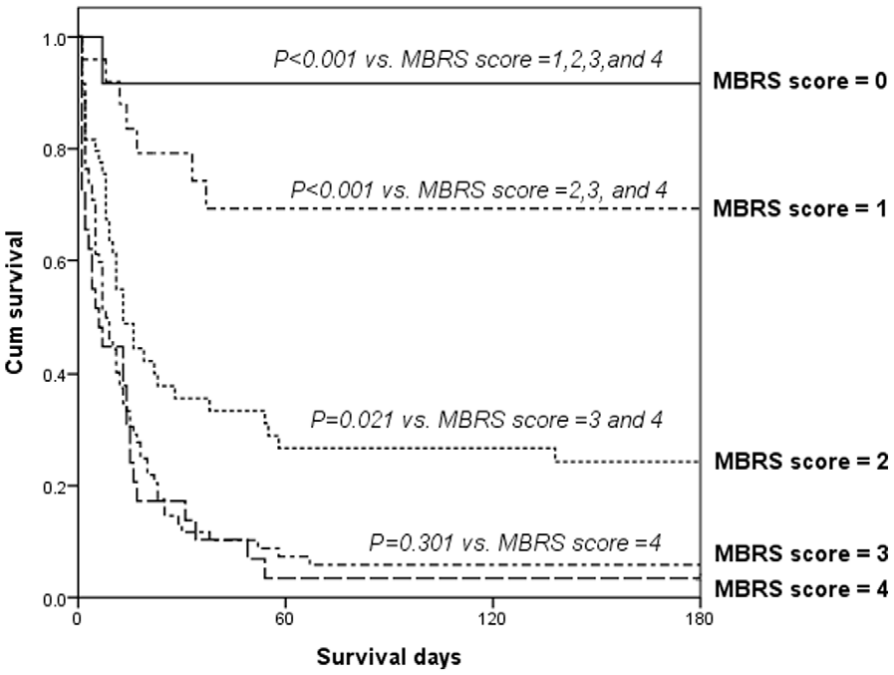
Survival Functions. Cumulative survival in 190 critically ill cirrhotic patients with acute kidney injury according to their MBRS (mean arterial pressure, bilirubin, respiratory failure and sepsis) score after the first day of admission to a specialized hepatogastroenterology intensive care unit.

The patient number and the in-hospital mortality rate calculated as per the stratification data of the MBRS scores has been listed in table 7. The in-hospital mortality rate was 8%, 26%, 72%, 93%, and 97% for MBRS scores of 0, 1, 2, 3, and 4, respectively (χ^2^ for trend, *p*<0.001). A progressive and significant increase in the mortality rate was observed to correlate with the increasing MBRS scores of the patients. With reference to an MBRS score of 0, the odds ratios for different MBRS scores were as follows: odds ratio for MBRS score of 1 = 3.85; odds ratio for MBRS score of 2 = 28.286; odds ratio for MBRS score of 3 = 147.74; and odds ratio for MBRS score of 4 = 308. Cumulative survival rates differed significantly (*p*<0.05) for patients with MBRS score of 0 and patients with MBRS scores of 1, 2, 3, and 4. The comparisons between patients with MBRS score of 1 and those with MBRS scores of 2, 3, and 4 and between patients with MBRS score of 2 and those with MBRS scores of 3, and 4 has been depicted in Figure 1.

**Table 7:** MBRS score for critically ill cirrhotic patients with AKI.

MBRS score	n	Hospital mortality (%)	Beta coefficient	Standard error	Odds rations (95%CI)	*p*
0	12	8	0	-	1 (reference)	-
1	27	26	1.348	1.133	3.850 (0.418–35.473)	NS (0.234)
2	50	72	3.342	1.091	28.286 (3.334–239.974)	0.002
3	72	93	4.993	1.143	147.740 (15.697–1384.178)	<0.001
4	29	97	5.730	1.458	308.000 (17.670–5368.505)	<0.001
Constant	-	-	-2.398	1.044	0.091	0.022

Abbreviation: AKI, acute kidney injury; MBRS, mean arterial pressure, bilirubin, respiratory failure and sepsis; CI, confidence intervals; NS, notsignificant.

## Discussion

In this study, the overall in-hospital mortality rate was 73.2%, which is consistent with the findings of previous reports and suggests that cirrhotic patients with AKI admitted to an ICU have an extremely poor prognosis [11], [24], [25]. This investigation showed that MBRS and APACHE III scores determined on the first day of admission to the ICU are significantly associated with in-hospital mortality in critically ill cirrhotic patients with AKI (Table 3). The MBRS score showed better discriminatory power than the Child-Pugh points, MELD, APACHE II, III, and SOFA scores (Table 4). The MBRS score had the best Youden index and the highest overall correctness of prediction (Table 6).

Our previous study showed the good discriminative power and independent predictive value of the MBRS scoring system in accurately predicting in-hospital mortality in critically ill cirrhotic patients with AKI [11]. The results of this study confirm these observations by showing that the MBRS score is a simple, reproducible, and easy-to-apply evaluation tool and has good prognostic value. This can help generate objective information for patients' families and physicians and supplement the judgments of clinical prognosis. Patients with cirrhosis are known to exhibit characteristic hyperdynamic circulation with secondary increase in heart rate and cardiac output and decrease in systemic vascular resistance, arterial blood pressure, and organ perfusion [26]–[28]. The fall in MAP resulted in glomerular blood flow decrease, and there was an autoregulation mechanism for keeping perfusion pressure which was achieved by pre-glomerular arteriole dilation until the cut-off point of MAP in 80 mmHg [29]. It was thought that MAP reflects not only the effective circulating volume caused by splanchnic vasodilation but also the instability of the hemodynamic system. Bilirubin level is a parameter reflecting both severity of an underlying liver illness and a superimposed liver injury caused by extrahepatic organ dysfunction [11]. Cirrhosis is associated with increased relative risk and death due to acute respiratory failure. In addition, cirrhotic patients requiring mechanical ventilation show an extremely poor prognosis [30]. Sepsis is a frequent cause of AKI and is associated with a poorer prognosis than that due to other causes. Patients with cirrhosis are susceptible to bacterial infections, which can lead to septic shock, metabolic acidosis, renal failure, hepatic encephalopathy, and decreased survival time [31]. The association of cirrhosis with such abnormalities makes the MBRS score an excellent tool for predicting in-hospital mortality in critically ill cirrhotic patients with AKI. Since no extrahepatic parameters are included in the determination of the Child-Pugh points, and no liver-specific prognostic factors are included in the determination of the APACHE II score, their discriminative powers are inferior to that of the MBRS score (Table 4).

This investigation has shown that APACHE III is an independent prognostic system for predicting in-hospital mortality in critically ill cirrhotic patients with AKI. The APACHE III system has been designed to increase the prediction accuracy of mortality in critically ill patients. A continuous weighing scheme for physiological variables, age, and comorbid conditions is used in this scoring system. However, the number of variables in this scoring system and their categorization has increased, and hence, enhancements in the statistical power increases the complexity of this system. Nevertheless, APACHE III is considered to be an economical scoring system to predict the severity of a disease and the probable mortality in patients [32].

In spite of the encouraging results observed in our study, several potential limitations in the study should also be considered. First, the study was conducted on patients from just 1 academic tertiary care medical center, which limits the generalization of our findings. Our results may be unsuitable for direct extrapolation to other hospitals with different patient populations. Second, the MBRS score is a specific scoring system developed only for cirrhotic patients with AKI who need intensive care support and not for the general ICU population. Third, we observed that hepatitis B viral infection (43%) was the leading cause of liver cirrhosis in patients and that a high proportion of patients had hepatoma (31%). This means that our results cannot be applied to the patients with liver disorders in North American and the European countries because liver diseases in these regions are largely because of hepatitis C viral infection or alcoholism. Fourth, the prognostic instruments were applied on patients already admitted to the specialized hepatogastroenterology ICU and were not used as a preadmission screening test; this may have skewed the results.

Fifth, defining baseline SCr as the first value measured during hospitalization might obscure the severity or even the presence of AKI. However, exactly true baseline SCr is not always available for all patients in clinical practice. Under various uncontrolled situation, choosing SCr established before admission as baseline value might run the risk to introduce some other biases and reduce the reproducibility of scoring systems. Due to above, many previous large studies also use admission SCr as baseline value to evaluate the impact of AKI on mortality in hospitalized patients [10], [25], [33]. As a matter of fact, cirrhotic patients who have stable renal function during hospitalization are thought to have a lower mortality rate, and such a relative low risk group is not our study target. Sixth, sequential measurement performed using these scoring systems (for example, daily, weekly) may reflect the dynamic aspects of the clinical diseases, and thus provide better information about the mortality risk in patients. Finally, the predictive accuracy of logistic regression models has its own limitations.

## Conclusion

In conclusion, this study showed the grave prognosis in critically ill cirrhotic patient with AKI. The analytical data also showed that the MBRS and APACHE III scoring systems were independent predictors of short-term treatment outcome in critically ill patients. We confirmed that the MBRS scorings system is an accurate, simple, easy-to-apply, reproducible, and economical system capable of providing an improved prediction of prognosis along with objective information for clinical decision making for treating a homogenous group of patients. On the basis of the observed results, we feel that critically ill cirrhotic patients with AKI who show high MBRS scores (≥2 points) should be prioritized for liver transplantation, if they are suitable candidates for undergoing a surgery.
